# Research progress on the role of ginsenoside Rd in central nervous system diseases

**DOI:** 10.4314/ahs.v24i4.41

**Published:** 2024-12

**Authors:** Xiaoyu Ma, Juejin Wang

**Affiliations:** 1 The Second Clinical School of Nanjing Medical University, Nanjing 211199, Jiangsu, China; 2 Department of Physiology, Nanjing Medical University, Nanjing 211199, Jiangsu, China

**Keywords:** Ginsenoside Rd, Stroke, Spinal cord injury, Alzheimer's disease, Parkinson's disease

## Abstract

Ginsenoside Rd (GSRd) is one of the rare saponin monomers extracted from ginseng. Most importantly, GSRd could effectively cross the intact blood-brain barrier (BBB). Studies have shown that it plays an important role in the treatment of neurological diseases such as ischemic stroke (IS), spinal cord injury (SCI), Alzheimer's disease (AD) and Parkinson's disease (PD). The results of these studies are of great significance for the clinical application of GSRd in the treatment in neurological diseases. This article reviewed the protective effects of GSRd in central nervous system diseases and analysed the related mechanism.

## Introduction

Ginsenosides are the main active substances in ginseng, American ginseng and Panax notoginseng in the genus Araliaceae. Ginsenoside Rd (GSRd) is one of the rare saponin monomers extracted from ginseng, which belongs to the diol type of dammarane-type ginsenosides, the molecular formula is C_48_H_82_O_18_•3H_2_O, the relative molecular mass is 1001, and some of its pharmacological effects are the same as other ginsenoside monomers^1^, ^2^. In recent years, in-depth research has found that compared with other saponins (ginsenoside Rb1, Rb2, Rc, Re, Rg1), GSRd has many characteristics that are different from other monomers. In terms of physical and chemical properties, GSRd has a small molecular weight, high fat solubility, and a long half-life in human plasma^2^, ^3^. Most importantly, GSRd could effectively cross the intact blood-brain barrier (BBB)^4^. GSRd has an important regulatory role in the cardiovascular, cerebrovascular and nervous systems. GSRd can improve microcirculation, reduce brain damage, resist atherosclerosis, enhance learning and memory, and delay body aging. This article reviewed the protective effects of GSRd in central nervous system diseases.

## Protective effect of GSRd on ischemic stroke (IS)

Ischemic stroke (IS) is an acute cerebrovascular disease in which the blood supply to the brain is insufficient due to the stenosis or occlusion of the blood supplying arteries of the brain, which resulting in damage and necrosis of brain tissue. The release of various types of reactive oxygen species and reactive nitrogen after cerebral ischemia can activate the oxidation of many macromolecules. The central nervous system is less resistant to oxidative stress and DNA damage. Degenerative diseases and trauma of the nervous system can cause a decrease in neurons and glial cells. Resistance to oxidative damage of nerve cells is an important pathway for ginsenosides to exert neuroprotective and neurotrophic effects^5^-^7^.

Ye et al. found that GSRd improved cerebral infarction and neurological outcomes in the middle cerebral artery occlusion (MCAO) rats^4^. Yang et al.^8^ used the MCAO rat model to study the neuroprotective function of GSRd on acute IS. The results showed that GSRd reduced mtDNA and nDNA damage, thereby helping to improve the survival rate of rats and the recovery of nerve function. Compared with MCAO rats not injected with GSRd, the NEIL1 and NEIL3 mRNA and protein levels of MCAO rats after injection were significantly up-regulated. The results indicated that that the neuroprotective function of GSRd for acute IS might be related to the up-regulation of NEIL1/3 expressions. Hu et al. found GSRd can protect against both neuronal cell death and inflammation after IS by inhibiting PARP-1 activity, sequential AIF translocation and NF-κB nuclear accumulation^9^. Besides, the neuroprotective mechanisms of GSRd were multiple including anti-inflammation, antioxidation, and antimitochondrial apoptosis via inhibition of poly(ADP-ribose) polymerase 1 (PARP-1)^9^-^13^. Xie et al. used MCAO and oxygen glucose deprivation (OGD)-induced ischemia-reperfusion injury (IRI) models to simulate in vivo or in vitro injury during cerebral ischemia. They pre-processed or post-processed the model with GSRd. The experimental results showed that GSRd significantly improved the behavioral score, infarct volume and viability of cultured neurons after ischemia, and GSRd inhibited the hyperphosphorylation of NR2B subunit and reduced the expression level of NR2B subunit in cell membrane. They proved that GSRd protects Sprague-Dawley (SD) rats and nerve cells from IRI by inhibiting the hyperphosphorylation of the NR2B subunit and reducing its expression in the cell membrane^14^.

Zhang et al. explored whether GSRd exerted its neuroprotective effect in IS as a new type of NMDAR blocker. Their whole-cell patch clamp results showed that GSRd dose-dependently reduced the NMDAR current of cultured rat cortical neurons by acting on the extra-synaptic NMDAR NR2b subunit. However, unexpectedly, cell transfection and radioligand binding assays showed that GSRd did not directly bind to the NMDAR channel. It inhibits the phosphorylation of NR2b at Ser-1303, which is the target of death-related protein kinase 1 (DAPK1). In addition, cell-based and cell-free enzymatic assays show that GSRd did not directly inhibit the activity of DAPK1, but blocks the activity of calcineurin, which is the key phosphatase for activating DAPK1. The inhibitory effect of cyclosporin A on calcineurin could mimic the effect of Rd and prevent neuronal damage caused by NMDA-, oxygen glucose deprivation- or transient ischemic stroke. Their study provided the first evidence that GSRd can reduce DAPK1-mediated phosphorylation of NR2b by attenuating calcineurin activity, thereby inhibiting the current and sequential excitotoxicity triggered by NMDAR^15^. Liu et al. studied the effect of GSRd on brain neurogenesis after IRI though male SD rats with MCAO followed by reperfusion. It was found that GSRd administration dose-dependently reduced the infarct size and nervous system score of IRI rats. GSRd significantly increased Akt phosphorylation in the ipsilateral hemisphere, and significantly increased the number of BrdU/DCX and Nestin/GFAP double-positive cells in the ischemic area. The above effects were partially blocked while Co-administration of I3 kinase inhibitor LY294002. Treatment with GSRd during reperfusion significantly increased the expression of VEGF and BDNF in PC12 cells with IRI. In addition, GSRd dose-dependently increased the phosphorylation of Akt and ERK, and significantly reduced PC12 cell apoptosis, which was blocked by the co-application of LY294002. The results indicated GSRd not only alleviated rat cerebral ischemia/reperfusion injury, but also increased VEGF and BDNF express and activate PI3K/Akt and ERK1/2 pathways. IS destroys the blood-brain barrier (BBB), causing brain edema and increasing the risk of intracranial hemorrhage. It has been found that proteasome inhibition protects the BBB from cerebral ischemia by inhibiting neuroinflammation-mediated matrix metalloproteinase-9 (MMP-9) activation. Zhang et al. showed that GSRd can inhibit proteasome-mediated inflammation and effectively treat IS. They found GSRd reduced the activity of the 20S proteasome in the cell-free test and inhibited the activity of the proteasome in the brain lysate after IS. GSRd administration inhibited NF-κB activity induced by IS and proteasome-mediated degradation of IκB. In addition, GSRd reduced the activity and level of MMP-9 (the downstream effector of NF-κB) and protected against BBB damage, which can be demonstrated by reducing Evan blue leakage and cerebral edema after cerebral IS. These data indicate that GSRd may reduce the pathogenesis of BBB injury by inhibiting proteasome activity and sequentially inhibiting the NF-κB/MMP-9 pathway^16^. The latest study by Yao et al. found that GSRd exerted an anti-pyroptotic effect to attenuate IS through the miR-139-5p/FoxO1/Keap1/Nrf2 axis in the male C57BL/6 mice MCAO/R model^17^.

The above research results show GSRd might possess great potential for clinical IS treatment. Accordingly, two clinical trials were performed to evaluate the treatment of GSRd for IS, in total, 190 patients in phase II and 390 patients in phase III were recruited. They were intravenously injected with GSRd (10, 20 mg) within 72 h after IS onset for 14 d. Clinical results showed that GSRd improved the National Institutes of Health Stroke Scale (NIHSS) of patients at 15 d, and did not significantly elevated mortality or adverse effects^18^. Hence, GSRd is one of the most potential drug candidates for IS.

## Protective effect of GSRd on spinal cord injury (SCI)

SCI is a widespread traumatic injury in the central nervous system and is the main cause of neurological dysfunction and death. The pathophysiology of SCI includes the primary injury during trauma, followed by a series of secondary molecular and cell damage, leading to loss of tissue and motor function^19^. The pathological mechanism of SCI is not clear, but it is generally believed that the primary mechanical shock will immediately affect the nerves. The tissue causes mechanical damage, leading to necrosis or cell death. The second injury occurs minutes to weeks after the initial injury and leads to a series of extracellular and intercellular events, including inflammatory cell infiltration, reactive gliosis, neuronal apoptosis and death, and oxidative stress^20^, ^21^. As the pathophysiological mechanism of SCI is not yet clear, the progress in the treatment of the disease is not satisfactory.

The effect of GSRd on SCI and its mechanism are still unclear. Cong et al. evaluated the neuroprotective effect of GSRd in a rat model of SCI. Rats in the SCI group underwent T8 laminectomy and spinal cord contusion. GSRd 12.5, 25, and 50 mg/kg were administered intraperitoneally 1 hour before surgery, once a day for 14 days. Dexamethasone 1 mg/kg was given as a positive control. Use the Basso-Beattie-Bresnahan scoring system to evaluate motor function. The results showed that GSRd 25 and 50 mg/kg significantly improved the motor function of rats after SCI, reduced tissue damage and increased the survival rate of neurons in the spinal cord. GSRd reduced MDA levels, increased GSH levels and SOD activity, reduced the production of pro-inflammatory cytokines and prevents cell apoptosis. The effect was comparable to dexamethasone. In addition, GSRd effectively inhibited the activation of MAPK signaling pathway induced by SCI, which may be related to the protective effect of GSRd on SCI^19^. MAPKs are evolutionarily conserved serine-threonine specific protein kinases that regulate a variety of cellular processes, including proliferation, differentiation, cell cycle, apoptosis, survival, inflammation, and cell death. In mammals, MAPKs include JNK, p38 and ERK. The MAPK pathway can be activated in various stress responses, including ROS-mediated oxidative stress and inflammatory cytokines^22^. An increase in phosphorylation levels of three MAPK proteins was found in SCI. The activation of MAPK may lead to cell apoptosis and worsen inflammation^23^. GSRd has a significant inhibitory effect on MAPK phosphorylation^24^. Zhou et al. studied the effect of GSRd on the integrity and redox balance of spinal cord mitochondria in vitro. The results showed that Ca^2+^ would dissipate the membrane potential, cause mitochondrial swelling and reduced the NAD(P)H matrix content, which were all attenuated by GSRd pretreatment in a dose-dependent manner. In contrast, GSRd could not inhibit Ca^2+^-induced mitochondrial hydrogen peroxide production. Western blot results showed that GSRd significantly increased the expression of p-Akt and p-ERK, but had no effect on the phosphorylation of PKC and p38. In addition, GSRd treatment significantly reduced Ca^2+^-induced cytochrome c release, which was partially reversed by antagonists of Akt and ERK, but not p-38 inhibitors. In addition, they also found that using GSRd in vivo pretreatment (10 and 50 mg/kg) to protect spinal cord mitochondria from Ca^2+^-induced mitochondrial membrane potential dissipation and cytochrome c release. They concluded that Rd regulates the formation of mitochondrial permeability transition pores and the release of cytochrome c through protein kinase-dependent mechanisms involving activation of Akt and ERK pathways in mitochondria^25^.

## Protective effect of GSRd on Alzheimer's disease (AD)

AD is a central nervous degenerative disease characterized by a large amount of β-amyloid proteins (Aβ) deposition in cells and hyperphosphorylation of tau protein, which can lead to memory loss and dementia^26^. Many researchers have revealed the important role of Aβ in AD. The deposition of Aβ in the brain of AD patients may play a role in immune stimulation, and inflammation is pathologically considered to play an important role in AD^27^. Liu et al. used Aβ precursor (APP) transgenic (Tg) mice to study the neuroprotective effect of GSRd on AD and its possible mechanism. It was found that GSRd could improve the learning and memory ability of APP Tg mice, possibly by inhibiting the transcriptional activity of NF-κB. As the activation of the NF-κB pathway is inhibited, the reduction of pro-inflammatory cytokines and the production of protective factors eventually increase.

In short, GSRd has neuroprotective effects on APP Tg mice and can be used as an alternative drug treatment for memory dysfunction in AD patients^28^.

Tau is a microtubule-associated protein expressed by the axons of central nervous system neurons, and its overexpression can induce neurodegenerative diseases^29^, ^30^. Tau phosphorylation is regulated by the balance of kinase and phosphatase activities. Research by Li et al. showed that GSRd could inhibit okadaic acid-induced tau phosphorylation in vivo and in vitro. They found that GSRd pretreatment inhibited tau phosphorylation at multiple sites in cultured cortical neurons treated with β-amyloid (Aβ), as well as in rats and transgenic mouse models. GiSRd not only reduced the increase in the expression of glycogen synthase kinase 3beta (GSK-3β) induced by Aβ, which is the most important kinase involved in tau phosphorylation, GSRd not only reduces Aβ-induced increase in the expression of glycogen synthase kinase 3beta (GSK-3β), but also inhibits its activity by increasing and decreasing its phosphorylation at Ser9 and Tyr216, respectively. In addition, GSRd enhanced the activity of protein phosphatase 2A (PP-2A), a key phosphatase involved in the dephosphorylation of tau. Finally, in vitro biochemical analysis showed that GSRd directly affects the activities of GSK-3β and PP-2A. Their results provide the first evidence that GSRd attenuates the pathological tau phosphorylation induced by Aβ through altering the functional balance of GSK-3β and PP-2A^31^, ^32^. Li et al. used 10 mg/kg GSRd to pretreat APP transgenic mice model for 6 months and found GSRd significantly decreased the hyperphosphorylated tau (S199/202, S396, and S404) protein in the olfactory bulb, spinal cord, and telencephalon via inhibiting the expression of GSK-3β/ Tyr216 and CDK5/P25^33^.

In addition, Yan et al. investigated whether GSRd could improve non-amyloidogenic pathway by activating estrogen receptor (ER) and found GSRd reduced cognitive and memory impairment in OVX rats, increased sAPPα levels and reduced extracellular Aβ. GSRd up-regulated the level of sAPPα in HT22, which was inhibited by MAPK and PI3K pathway inhibitors. In addition, estrogen receptor inhibitors prevent GSRd from triggering the release of sAPPα and the activation of MAPK and PI3K pathways. GSRd increased the expression of α-secretase and sAPPα, but decreased the expression of β-secretase and Aβ.

In addition, GS-Rd promoted the phosphorylation of estrogen receptor alpha at residue Ser118. They proved that GSRd can regulate MAPK/ERK and PI3K/AKT signaling pathways through estrogen receptor α (ERα), decrease Aβ expression by suppressing β-secretase, and increase SAPPα expression by promoting α-secretase in the treatment of neurological diseases^34^.

## Protective effect of GSRd on Parkinson's disease (PD)

PD is the second most common neurological disease affecting millions of people worldwide^35^. PD is a progressive disease characterized by the loss of neurons in the substantia nigra striatum^36^. Although the symptoms are already clear, the exact etiology, especially the mechanism of PD substantia nigra dopaminergic neuron degeneration is still unknown^37^. The protective effect of GSRd on PD is mainly concentrated in in vitro cell research.

Carbon tetrachloride (CCl4) can produce neurotoxic effects when present as an environmental pollutant. As a model compound, it can be used to study the effect on dopaminergic nerve cells in the primary substantia nigra striatum. Zhang et al. used CCl4 to prepare PD cell models in vitro and studied the neuroprotective potential of GSRd and GSRe. It was found that, compared with the untreated control culture, CCl4 (2.5 mM on day 12 in vitro for 48 hours) significantly reduced the number of tyrosine hydroxylase (TH+) cells by 51% and reduced the length of neuritis and leaded to truncated degradation of cell morphology. GSRd and GSRe (10 µM) strongly reduced cell loss and degradation, and significantly protected the process length and number of neurites of TH+ cells. CCl4 exposure reduced the antioxidant and anti-inflammatory potential of the cell supernatant. The addition of GS inhibited oxidative stress and inflammation. Therefore, the neuroprotective effects of GSRd and GSRe depend at least in part on reducing oxidative stress and anti-inflammatory effects. Liu et al. investigated whether GSRd can play a beneficial role in an in vitro experimental PPD model, in which SH-SY5Y cells were damaged by 1-methyl-4-phenylpyridine (MPP+), which is a classic Parkinson's toxin 1-methyl-4-phenyl-1,2,3,6-tetrahydropyridine (MPTP) product. Their results found that 1 µM and 10 µM GSRd significantly attenuated cell death induced by MPP+. This protective effect may be attributed to its ability to reduce intracellular reactive oxygen levels, enhance antioxidant enzyme activity, maintain respiratory complex I activity, stabilize mitochondrial membrane potential, and increase intracellular ATP levels. In addition, the PI3K/Akt survival signaling pathway was also involved in the protection of GSRd. Finally, they used an in vivo mouse PD model and found that GSRd significantly reversed the MPTP-induced loss of tyrosine hydroxylase-positive cells in the substantia nigra. Their research results show that GSRd shows significant neuroprotective effects on experimental PD models, which may involve its antioxidant effects and protection of mitochondrial function^38^. In PD, clinical and experimental evidence indicates that neuroinflammatory changes in cytokines caused by activation of microglia lead to neuronal death. Neuroinflammation of dopaminergic neurons can be caused by lipopolysaccharide (LPS) exposure. Lin et al. experimentally used LPS to induce inflammation of midbrain dopamine nerve cells, and found that adding LPS (100 microg/ml) to the primary midbrain dopamine nerve cell culture can cause 30-50% of dendritic process loss, perinuclear changes, TH immune response (TH+) cell shrinkage and neuronal cell loss. iNOS activity and prostaglandin E2 concentration increased in a dose-dependent manner. At the same time, adding GSRd can counteract the neurodegeneration induced by LPS. Moreover, the neuroprotective effect of ginsenoside Rd was not selective for dopaminergic neurons. The anti-inflammatory effect of GSRd may be achieved by reducing the formation of NO and the synthesis of PGE2. Therefore, the protective mechanism of GSRd may involve interference with the expression of iNOS and COX-2.

In addition, GSRd has also been reported to play a role in other neurological diseases. Liu et al. reported that GSRd is able to inhibit glioblastoma cell proliferation by up-regulating the expression of miR-144-5p^39^. The latest study by Li et al. found that GSRd can exhibit significant antidepressant-like effect via HIF-1α signaling pathway^40^.

In summary, GSRd is a promising natural neuroprotective agent, the mechanism of the neuroprotective effect of GSRd is related to anti-inflammatory, anti-oxidant, anti-apoptosis, protection of the blood-brain barrier, promotion of nerve regeneration, anti-glutamate excitotoxicity, DNA repair, mitochondrial protection, regulation of Ca2+ and neurological factors, which is related to the regulation of NF-κB, PI3K/AKT, MAP/ERK, PARP, DAPK1 signaling pathways. The current research results are mostly based on animal experiments. In the future, the clinical verification of the neuroprotective effect of GSRd should be carried out to lay the foundation for development of new drugs.
